# Influence of additional intra-articular arthroscopic visualization on surgeons’ assessment of reduction adequacy in distal radius fractures: A survey-based observational study

**DOI:** 10.1097/MD.0000000000051447

**Published:** 2026-10-30

**Authors:** Murat Gök, Mücahit Çelik, Özgün Karakuş

**Affiliations:** a Department of Orthopaedics and Traumatology, University of Health Sciences, Balıkesir Atatürk City Hospital, Balıkesir, Turkey; b Department of Orthopaedics and Traumatology, Balıkesir Atatürk City Hospital, Balıkesir, Turkey.

**Keywords:** distal radius fracture, fluoroscopy, intra-articular fracture, reduction adequacy, surgical decision-making, wrist arthroscopy

## Abstract

This study investigates whether additional intra-articular arthroscopic visualization alters experienced orthopedic surgeons’ assessment of reduction adequacy and their image-based consideration of revision during intraoperative evaluation of intra-articular distal radius fractures compared with fluoroscopic assessment alone. Rather than evaluating clinical outcomes, the study aimed to determine whether direct visualization of the articular surface influences surgeons’ interpretation of the conventional fluoroscopic reduction criteria. Intraoperative fluoroscopic and corresponding wrist arthroscopic images from 2 purposively selected, surgically treated intra-articular distal radius fractures (AO/ASIF type 23-C2) were evaluated by 196 orthopedic surgeons with at least 10 years of post-residency clinical experience. For each case, participants first assessed fluoroscopic images and answered standardized questions regarding reduction adequacy and whether they would consider revision surgery. The same questions were subsequently answered after reviewing the corresponding arthroscopic images. Paired categorical responses were compared using the McNemar test, with statistical significance set at *P* < .05. For case 1, the proportion of surgeons considering the reduction acceptable decreased from 94.4% (185/196) after fluoroscopic evaluation to 10.2% (20/196) following arthroscopic assessment (*P* < .001). Arthroscopic evaluation also significantly increased consideration of revision surgery (*P* < .001). In case 2, reduction acceptability did not differ between imaging modalities (*P* = 1.00); however, arthroscopic visualization significantly increased the proportion of surgeons indicating that they would consider revision surgery (*P* < .001). In this exploratory survey based on 2 purposively selected intra-articular distal radius fracture cases, additional static arthroscopic visualization altered experienced surgeons’ image-based assessments of reduction adequacy and their hypothetical consideration of revision. These findings reflect changes in interpretation after additional intra-articular visual information and should not be considered evidence of diagnostic superiority, improved anatomical reduction, or clinical benefit.

## 1. Introduction

Distal radius fractures are among the most frequently encountered injuries in orthopedic practice. Treatment options range from conservative management to surgical intervention depending on fracture characteristics, patient-related factors, and associated injuries. Intra-articular fractures present a particular challenge because restoration of articular congruity is considered essential for optimizing functional outcomes and reducing the risk of posttraumatic arthritis. Consequently, accurate intraoperative assessment of reduction quality plays a pivotal role in surgical decision-making.^[1–4]^

Several classification systems have been developed to describe distal radius fractures. Among these, the Arbeitsgemeinschaft für Osteosynthesefragen/Association for the Study of Internal Fixation (AO/ASIF) classification is widely used because of its reproducibility and applicability in both clinical practice and research.**^[5–8]^**

Regardless of the classification system, the primary objective of surgical treatment is to restore anatomical radiocarpal congruity and permit early functional recovery.^[9,10]^

Radiographic parameters are routinely used to evaluate reduction quality. Commonly accepted criteria include <5 mm of radial shortening, <5° loss of radial inclination, dorsal angulation of <5°, and an intra-articular step-off of <2 mm.^[11,12]^ Among these parameters, the 2-mm articular step-off threshold is particularly important because incongruities exceeding this value have been associated with an increased risk of post-traumatic arthritis.^[13]^ Consequently, intraoperative recognition of this threshold frequently determines whether a reduction is considered acceptable.

Fluoroscopy remains the standard intraoperative imaging modality for assessing fracture reduction and implant position. Nevertheless, as a 2-dimensional projection technique, fluoroscopy has inherent limitations in visualizing complex articular surfaces and detecting subtle residual incongruities.^[14–16]^ Wrist arthroscopy provides direct magnified visualization of the articular surface and may reveal residual step-offs or associated intra-articular lesions that are not readily appreciated on conventional fluoroscopic imaging.^[17,18]^

Previous studies evaluating arthroscopy-assisted fixation have mainly focused on radiographic reduction quality or postoperative clinical outcomes, with inconsistent results regarding the additional value of arthroscopy.^[17,18]^ However, considerably less attention has been paid to whether the additional intra-articular information provided by arthroscopy influences surgeons’ interpretation of the conventional fluoroscopic reduction criteria during intraoperative assessment.

The present study was designed to address this specific question. Rather than evaluating the clinical superiority of wrist arthroscopy or postoperative patient outcomes, we investigated whether additional intra-articular arthroscopic visualization changes experienced orthopedic surgeons’ assessment of reduction adequacy and their consideration of revision when evaluating the same intraoperative reduction. We hypothesized that direct visualization of the articular surface would alter surgeons’ interpretation of reduction acceptability despite identical fluoroscopic findings.

## 2. Materials and methods

### 2.1. Study design

This cross-sectional, observational, survey-based study was designed to investigate whether additional intra-articular arthroscopic visualization influences experienced orthopedic surgeons’ assessment of reduction adequacy during intraoperative evaluation of intra-articular distal radius fractures. Rather than evaluating clinical outcomes or the diagnostic accuracy of either imaging modality, the study focused on changes in surgeons’ interpretation of the same intraoperative reduction following sequential fluoroscopic and arthroscopic assessment.

### 2.2. Case selection and imaging

Two surgically treated intra-articular distal radius fracture cases were purposively selected from the institutional archive. Eligibility for consideration required the availability of both postfixation intraoperative fluoroscopic images and corresponding wrist arthroscopic images obtained during the same surgical procedure. Both operations were performed by the same surgeon, both fractures were classified as AO/ASIF type 23-C2 fractures, and both were treated with volar plate fixation.^[7,8]^ The cases were not selected consecutively or randomly.

The selection was intentionally illustrative and was not based on the severity of the preoperative radiographic appearance. Based on the operating surgeon’s intraoperative fluoroscopic assessment, case 1 represented a relatively more satisfactory reduction appearance, whereas case 2 represented a relatively less satisfactory reduction appearance. These 2 contrasting fluoroscopic scenarios were deliberately selected to explore whether the effect of additional arthroscopic visualization on surgeons’ assessments varied according to the apparent quality of reduction on fluoroscopy.

The study was not designed to determine whether arthroscopy is more accurate than fluoroscopy or to evaluate whether arthroscopy is particularly beneficial in fractures with more severe preoperative displacement. Rather, it examined whether direct visualization of the articular surface altered surgeons’ interpretation of commonly accepted fluoroscopic reduction criteria, including the 2-mm intra-articular step-off threshold. To improve transparency regarding baseline fracture morphology, the preoperative anteroposterior, and lateral radiographs of both cases are provided in Figure S1, Supplemental Digital Content 1. The postfixation fluoroscopic and corresponding arthroscopic images are presented in Figs. 1 and 2.

**Figure 1.**
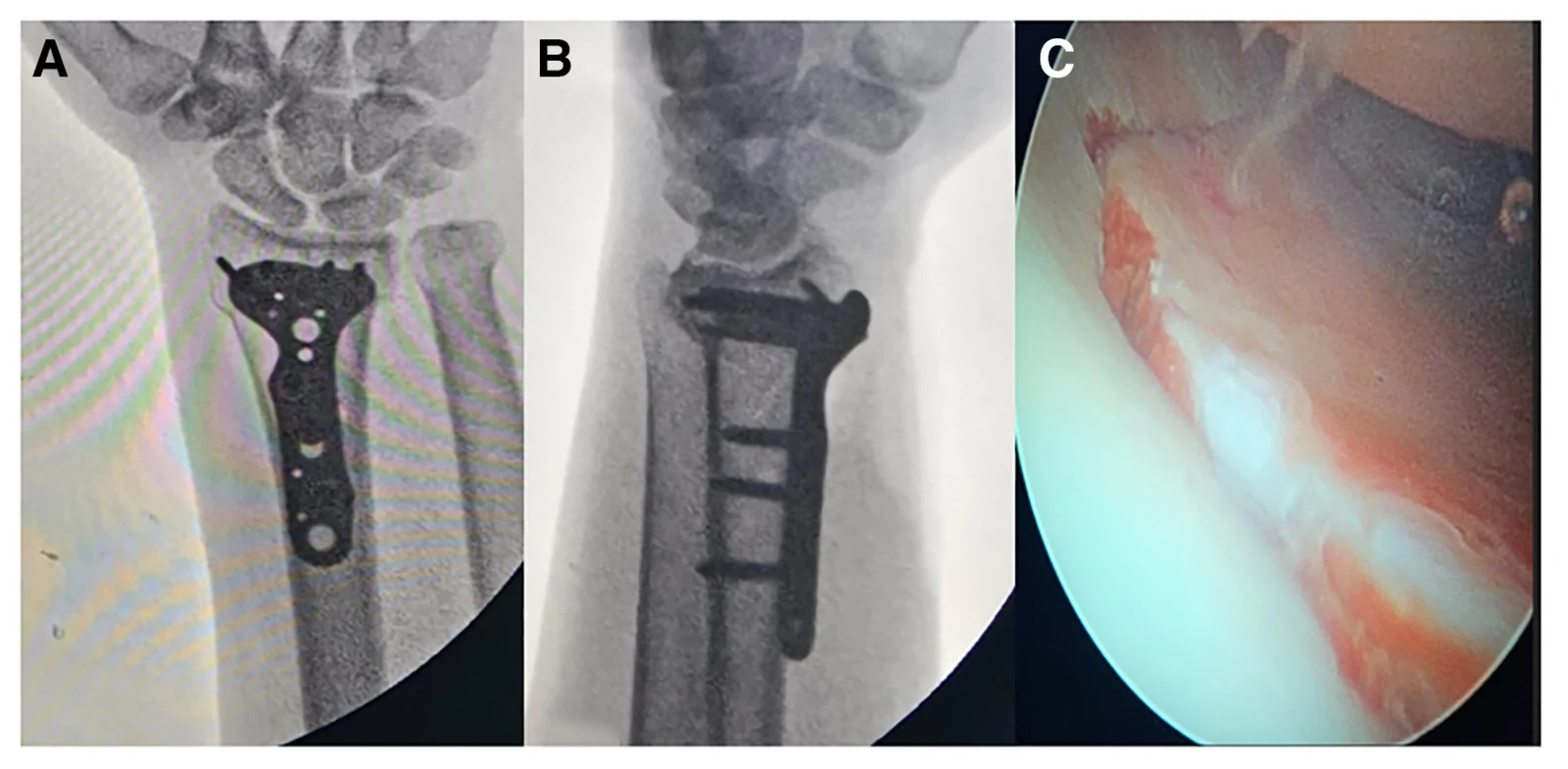
Case 1 images: (A) Postfixation intraoperative posteroanterior fluoroscopic image following osteosynthesis of a distal radius fracture; (B) postfixation intraoperative lateral fluoroscopic image; and (C) intra-articular wrist arthroscopic image obtained after osteosynthesis.

**Figure 2.**
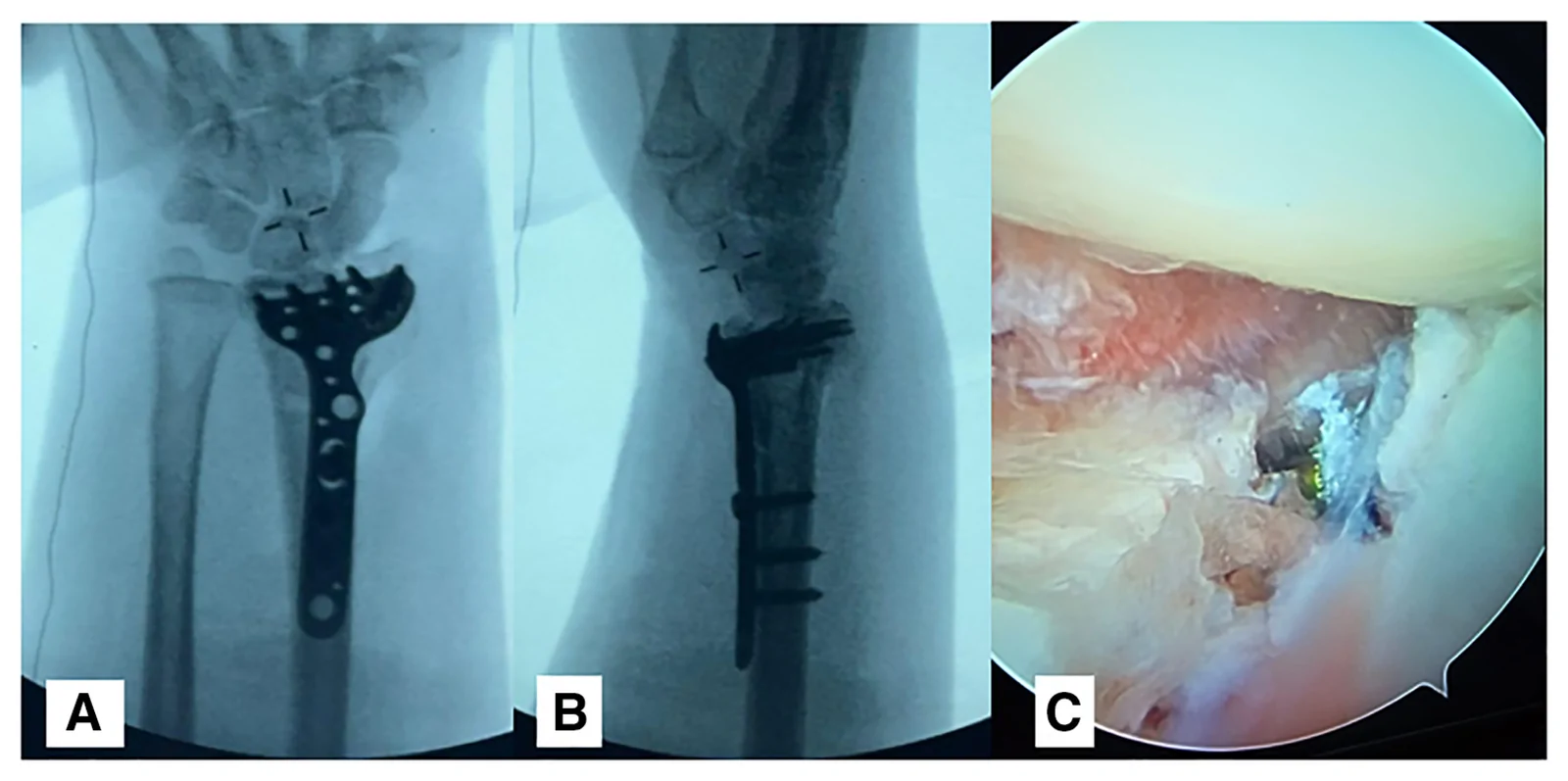
Case 2 images: (A) Post-fixation intraoperative posteroanterior fluoroscopic image following osteosynthesis of a distal radius fracture; (B) postfixation intraoperative lateral fluoroscopic image; and (C) intra-articular wrist arthroscopic image obtained after osteosynthesis.

### 2.3. Participants

A total of 196 orthopedic and traumatology surgeons participated in the survey. Eligibility required at least 10 years of post-residency clinical experience and self-reported performance of at least 20 distal radius fracture operations annually. The annual case-volume threshold was used as a pragmatic eligibility criterion to confirm active involvement in distal radius fracture surgery and was not intended to represent a validated definition of surgical expertise or proficiency in wrist arthroscopy. All participants independently evaluated the images after receiving standardized study instructions.

The survey did not collect participants’ exact annual operative volume beyond confirmation of the eligibility threshold. Information regarding age, institutional affiliation, subspecialty training, dedicated hand or wrist fellowship training, and specific wrist arthroscopy experience was also not collected. Consequently, subgroup analyses according to wrist arthroscopy experience could not be performed.

### 2.4. Evaluation procedure

Each participant independently evaluated both cases using a standardized 2-stage assessment presented in a fixed sequence.

### 2.5. Stage 1: Fluoroscopic assessment

During stage 1, participants were shown 2 static postfixation fluoroscopic images of each case, consisting of standard posteroanterior and lateral projections. Based exclusively on these fluoroscopic images, they answered 2 intentionally concise binary questions:

- Is the reduction adequate? (ADEQUATE/ INADEQUATE)
- Would you consider revision surgery? (YES/NO)

### 2.6. Stage 2: Arthroscopic assessment

During stage 2, participants were shown the corresponding static intra-articular arthroscopic image obtained from the same case and were asked the same 2 questions again. No dynamic fluoroscopic sequences, additional fluoroscopic projections, arthroscopic video recordings, or tactile probing information were provided.

The fixed fluoroscopy-to-arthroscopy sequence was selected because the study specifically aimed to evaluate whether the addition of direct intra-articular visual information altered surgeons’ assessments after they had evaluated the reduction using standard fluoroscopic images. The study was not designed as a randomized diagnostic-accuracy comparison between fluoroscopy and arthroscopy.

Participants were unable to return to or modify their previous responses. The revision item was intended to measure a hypothetical inclination to consider revision based on the presented images rather than a definitive clinical decision to perform revision surgery.

Responses were electronically recorded and analyzed as paired categorical data for each participant.

### 2.7. Statistical analysis

Because each participant evaluated both fluoroscopic and arthroscopic images from the same case, paired nominal data were obtained. Differences between fluoroscopic and arthroscopic assessments of reduction adequacy and revision-related responses were analyzed using the McNemar test. Statistical significance was defined as a 2-sided *P* value < .05.

To better quantify the magnitude of decision changes, absolute frequencies, percentages, and paired response distributions were reported for each comparison. Corresponding 95% confidence intervals and effect size estimates were additionally calculated where appropriate.

All statistical analyses were performed using IBM SPSS Statistics for Windows (version 26.0, IBM Corp.).

### 2.8. Ethical approval and informed consent

This study was conducted in accordance with the principles of the Declaration of Helsinki. Ethical approval was obtained from the Balıkesir Atatürk City Hospital Scientific Research Ethics Committee (Decision No. 2024/10/55; October 24, 2024).

All participating surgeons provided informed consent before participation. The intraoperative fluoroscopic and arthroscopic images were fully anonymized, and the Ethics Committee waived the requirement for additional patient consent for publication of anonymized intraoperative images.

## 3. Results

### 3.1. Case 1 – reduction acceptability

For case 1, reduction was considered acceptable in 185 of 196 evaluations (94.4%) based on fluoroscopic assessment and in 20 of 196 evaluations (10.2%) following arthroscopic assessment. McNemar analysis demonstrated a significant difference between the 2 assessment modalities (*P* < .001). Of the 185 surgeons who initially considered the reduction acceptable on fluoroscopy, 165 (89.2%) changed their assessment to unacceptable after reviewing the arthroscopic images, whereas no participant changed from an unacceptable to an acceptable assessment. The paired distribution of responses is presented in Table 1.

**Table 1:** Paired comparison of reduction acceptability between fluoroscopy and wrist arthroscopy in case 1.

Fluoroscopy assessment	Arthroscopy acceptable	Arthroscopy unacceptable	Total
Acceptable	20	165	185
Unacceptable	0	10	10
Total	20	175	196

CI = confidence interval, OR = odds ratio.

McNemar exact test: *P* = 4.28 × 10^−50^.

Discordant pairs: 165/196 (84.2%).

Matched odds ratio: ∞.

Exact 95% CI for matched OR: 44.23–∞.

### 3.2. Case 2 – reduction acceptability

For case 2, all 196 participants considered the reduction unacceptable on both fluoroscopic and arthroscopic assessment. Consequently, no changes in reduction acceptability were observed (*P* = 1.00). The paired response distribution is shown in Table 2.

**Table 2:** Paired comparison of reduction acceptability between fluoroscopy and wrist arthroscopy in case 2.

Fluoroscopy assessment	Arthroscopy acceptable	Arthroscopy unacceptable	Total
Acceptable	0	0	0
Unacceptable	0	196	196
Total	0	196	196

McNemar exact test: *P* = 1.00.

Discordant pairs: 0/196 (0.0%).

Matched odds ratio: Not applicable.

#### 3.2.1. Case 1 – consideration of revision

For case 1, revision was considered by 5 of 196 participants (2.6%) following fluoroscopic assessment and by 20 of 196 participants (10.2%) following arthroscopic assessment (*P* < .001). Fifteen participants changed from not considering revision to considering revision after reviewing the arthroscopic images, whereas no participant changed from considering revision to not considering revision.

#### 3.2.2. Case 2 – consideration of revision

For case 2, the number of participants indicating that they would consider revision increased from 130 of 196 (66.3%) following fluoroscopic assessment to 190 of 196 (96.9%) following arthroscopic assessment (*P* < .001). Sixty participants changed from not considering revision to considering revision following arthroscopic assessment, whereas no participant changed from considering revision to not considering revision.

### 3.3. Summary of decision changes

The overall decision-change rate for reduction acceptability was 84.2% in case 1 and 0% in case 2. For revision-related responses, corresponding change rates were 7.7% and 30.6%, respectively. A summary of paired changes in reduction acceptability and revision-related responses is presented in Table 3.

**Table 3:** Summary of paired changes in reduction assessment and revision decisions following arthroscopic evaluation.

Outcome	Case	Fluoroscopy, n (%)	Arthroscopy, n (%)	Changed after arthroscopy, n (%)	McNemar exact *P* value	Matched OR (95% CI)
Reduction considered acceptable	Case 1	185 (94.4)	20 (10.2)	165 (84.2)	4.28 × 10^−50^	∞ (44.23–∞)
Reduction considered acceptable	Case 2	0 (0.0)	0 (0.0)	0 (0.0)	1.00	Not applicable
Revision considered	Case 1	5 (2.6)	20 (10.2)	15 (7.7)	6.10 × 10^−5^	∞ (3.59–∞)
Revision considered	Case 2	130 (66.3)	190 (96.9)	60 (30.6)	1.73 × 10^−18^	∞ (15.77–∞)

CI = confidence interval, OR = odds ratio.

## 4. Discussion and conclusion

The principal finding of the present study is not that wrist arthroscopy is superior to fluoroscopy, but that additional intra-articular visualization substantially altered experienced surgeons’ assessment of reduction adequacy when evaluating the same intraoperative reduction. In case 1, a reduction considered acceptable by 94.4% of participants based on fluoroscopic assessment was considered acceptable by only 10.2% after arthroscopic evaluation. Rather than demonstrating the superiority of 1 imaging modality over another, these findings indicate that the interpretation of the conventional fluoroscopic reduction criteria – particularly the widely accepted 2-mm intra-articular step-off threshold – may change when direct visualization of the articular surface becomes available. Accordingly, the present study should be interpreted as demonstrating a change in intraoperative assessment rather than establishing the clinical superiority of wrist arthroscopy.^[13–15]^

The present findings should be interpreted within the context of the conventional intraoperative reduction criteria rather than as evidence supporting 1 imaging modality over another. Restoration of an intra-articular step-off of <2 mm remains one of the most widely accepted criteria for successful reduction because residual incongruity beyond this threshold has been associated with posttraumatic degenerative changes.^[12,19]^ However, the present study suggests that applying this criterion may depend not only on the threshold itself but also on the imaging modality used to assess it. When the identical reduction was evaluated with additional intra-articular visualization, a substantial proportion of surgeons modified their assessment of reduction adequacy. These findings do not establish that arthroscopy provides the true anatomical reference standard; rather, they demonstrate that direct visualization of the articular surface may alter surgeons’ interpretation of the conventional fluoroscopic reduction criteria. This distinction is important because the objective of the present study was to evaluate changes in intraoperative assessment rather than the diagnostic accuracy of arthroscopy.

The observed changes should therefore be interpreted as changes in surgeons’ responses after receiving additional static intra-articular visual information, rather than as evidence that arthroscopy is diagnostically superior to a complete dynamic fluoroscopic examination. The findings illustrate the difficulty of applying commonly accepted articular reduction criteria, including the approximately 2-mm step-off threshold, using a limited set of standard fluoroscopic projections alone.

Previous studies investigating arthroscopy-assisted fixation have primarily evaluated radiographic reduction quality, postoperative imaging, or functional outcomes, and their conclusions remain inconsistent. While some authors have reported improved restoration of articular congruity with arthroscopy-assisted reduction,^[18,20]^ systematic reviews have not demonstrated consistent evidence that arthroscopy improves radiographic or functional outcomes compared with fluoroscopy alone, although its value in identifying associated ligamentous and soft-tissue injuries has been consistently recognized.^[8,10]^ One potential explanation for these conflicting findings is that reduction quality in most previous studies was determined by the operating surgeon, making it difficult to distinguish between the technical success of the procedure and the surgeon subjective interpretation of reduction adequacy.^[21]^

The present study was designed to address a different question. Rather than comparing clinical outcomes or technical performance, we evaluated whether the same intraoperative reduction would be interpreted differently when additional intra-articular visualization became available. By using paired assessments from the same group of experienced orthopedic surgeons, each participant served as his or her own control, thereby allowing the observed differences to reflect changes in interpretation rather than differences between observers.^[22]^ Accordingly, the present findings should be viewed as evidence that additional intra-articular information may influence intraoperative assessment, rather than as evidence that arthroscopy itself improves reduction quality or patient outcomes.

The different response patterns observed between the 2 cases further support the interpretation that the present study evaluated changes in surgeons’ assessment rather than the diagnostic performance of arthroscopy. In case 1, arthroscopic visualization markedly altered the perceived adequacy of reduction, whereas in case 2 no change was observed in reduction acceptability despite a substantial increase in participants indicating that they would consider revision. These findings suggest that the additional information obtained through arthroscopy may influence different aspects of intraoperative decision-making depending on the specific intra-articular findings present in an individual case.

Arthroscopy-assisted techniques have been described for assessing and addressing residual articular incongruity during distal radius fracture fixation.^[23]^ Arthroscopic studies have also documented associated intra-articular soft-tissue injuries in distal radius fractures.^[24]^ The revision-related responses should be interpreted as image-based hypothetical inclinations rather than definitive decisions to undertake revision surgery. The intentionally concise binary question was used to capture whether the addition of arthroscopic visualization changed participants’ immediate consideration of revision. In actual clinical practice, however, the decision to revise a fixation construct is considerably more complex and depends on patient age, functional demand, hand dominance, occupation, bone quality, comorbidities, anesthetic considerations, and the anticipated morbidity and technical extent of the revision procedure. Accordingly, the observed changes indicate that additional intra-articular visual information influenced participants’ willingness to consider revision, but they do not establish that revision would have been performed in a real operative setting.

Several limitations of the present study should be acknowledged. First, although the survey included paired assessments from 196 experienced orthopedic surgeons, only 2 purposively selected fracture cases were evaluated. The large number of evaluators does not compensate for the limited number and diversity of clinical cases. Because the 2 cases were intentionally selected to represent relatively different fluoroscopic reduction appearances, the observed response patterns may have been influenced by case-specific fracture morphology and reduction characteristics. Moreover, both cases involved the same AO/ASIF fracture pattern and were treated with volar plate fixation. Fracture severity, fracture morphology, and fixation strategy may influence surgeons’ tolerance for residual articular incongruity, their threshold for considering revision, and the technical feasibility of further correction. Because these variables were not represented across multiple cases or fixation methods, the present findings should be interpreted specifically within the clinical context of AO/ASIF type 23-C2 fractures treated with volar plate fixation. Accordingly, the findings cannot be generalized to the broader spectrum of intra-articular distal radius fractures and should not be interpreted as estimates of the prevalence or expected direction of decision changes in routine clinical practice. Rather, they should be regarded as exploratory, hypothesis-generating observations demonstrating that additional intra-articular visualization can alter surgeons’ interpretation in selected cases.

Second, the survey was based on static images and therefore did not reproduce the complete intraoperative assessment available during actual surgery. In clinical practice, fluoroscopy may include dynamic wrist positioning, multiple or tangential projections, and repeated C-arm adjustments, while arthroscopy additionally permits real-time visualization and tactile assessment with a probe. None of these dynamic or tactile elements was available to the participants. Consequently, the observed magnitude and direction of decision change reflect responses to the selected static images and may differ from those obtained during a dynamic intraoperative examination, in which surgeons can optimize fluoroscopic projections, assess findings from multiple angles, and perform real-time arthroscopic probing.

Third, all participants evaluated the fluoroscopic images before viewing the arthroscopic image, and the sequence was not randomized. This fixed order was integral to the study question, which focused on whether adding intra-articular visual information after standard fluoroscopic assessment altered surgeons’ responses. Nevertheless, an order, priming, or sequential learning effect cannot be excluded, even though participants were unable to return to or modify their earlier answers. Therefore, the study design cannot completely separate the effect of the additional imaging modality from the effect of repeated assessment or sequential reconsideration. Future studies using randomized or crossover image-presentation designs would help distinguish the effect of imaging modality from the effect of presentation order.

Fourth, postoperative computed tomography was not used to validate the anatomical accuracy of either imaging modality. Therefore, the present study was not designed to determine whether arthroscopic assessment more accurately reflects true osseous reduction, but rather whether additional intra-articular visualization influences surgeons’ interpretation of conventional fluoroscopic reduction criteria. Fifth, no postoperative functional or radiographic outcome data were available. Consequently, the observed changes in assessment should not be interpreted as evidence that altered intraoperative decisions necessarily translate into improved clinical outcomes.

Although all participants had at least 10 years of post-residency clinical experience and reported performing at least 20 distal radius fracture operations annually, these criteria establish active experience in fracture surgery rather than proficiency in wrist arthroscopy. Specific information regarding wrist arthroscopy training, fellowship background, and annual wrist arthroscopy volume was not collected. Arthroscopic image interpretation may be influenced by procedure-specific training and experience; therefore, unmeasured differences in wrist arthroscopy experience represent a potential confounding factor. Because these data were unavailable, we could not determine whether the observed changes in assessment differed between surgeons with greater and lesser wrist arthroscopy experience.

The binary revision question represents an additional limitation. Participants were asked only whether they would consider revision surgery, without being provided with patient-specific clinical characteristics or a standardized definition of what the revision would entail. Moreover, the forced yes/no response format did not permit intermediate responses such as “uncertain,” “additional imaging required,” or “dependent on patient factors.” This simplified format was useful for comparing paired changes in immediate image-based responses but does not capture the multifactorial nature of actual intraoperative decision-making. The reported revision-related findings should therefore not be interpreted as actual revision indications or as evidence that the participants would have undertaken revision surgery in clinical practice.

Future studies should evaluate a larger and more diverse series of consecutively enrolled intra-articular distal radius fractures, including different fracture patterns, levels of fracture severity and reduction quality, and fixation methods. They should also prospectively record participants’ subspecialty training, annual distal radius fracture volume, and wrist arthroscopy experience and use randomized or crossover image-presentation designs. Such studies would help determine how frequently additional arthroscopic visualization alters surgeons’ assessments and whether the magnitude or direction of this change varies according to fracture morphology, apparent fluoroscopic reduction quality, presentation order, or surgeon experience.

## 5. Conclusion

In this exploratory survey based on 2 purposively selected intra-articular distal radius fracture cases, additional arthroscopic visualization altered experienced surgeons’ assessments of reduction adequacy and their consideration of revision when evaluating the same intraoperative reductions. These findings reflect changes in image interpretation and should not be interpreted as evidence of the superiority of arthroscopy, improved anatomical reduction, or clinical benefit. Because the observed response patterns may be case-specific and were derived exclusively from 2 AO/ASIF type 23-C2 fractures treated with volar plate fixation, they cannot be generalized to other fracture patterns, levels of fracture severity, or fixation methods. Larger studies incorporating a more diverse and consecutively selected series of cases are required to determine the reproducibility and clinical significance of these observations.

## Author contributions

**Data curation:** Murat Gök.

**Formal analysis:** Murat Gök.

**Resources:** Murat Gök, Mücahit Çelik, Özgün Karakuş.

**Visualization:** Murat Gök.

**Methodology:** Özgün Karakuş.

**Validation:** Özgün Karakuş

**Writing – original draft:** Mücahit Çelik.

**Writing – review & editing:** Mücahit Çelik.



## References

[1] Van Schoonhoven J. Distal radius fractures-concepts and treatment reality. Unfallchirurgie (Heidelb). 2024;127:411–2. 10.1007/s00113-024-01438-w 38805062

[2] Slutsky DJ Herman M. Rehabilitation of distal radius fractures: a biomechanical guide. Hand Clin. 2005;21:455–68. 10.1016/j.hcl.2005.01.004 16039457

[3] de Bruijn MAN van Ginkel LA Boersma EZ. The past, present and future of the conservative treatment of distal radius fractures. Injury. 2023;54(Suppl 5):110930. 10.1016/j.injury.2023.110930 37923503

[4] Ilyas AM Jupiter JB. Distal radius fractures--classification of treatment and indications for surgery. Hand Clin. 2010;26:37–42. 10.1016/j.hcl.2009.08.003 20006242

[5] Jakob M Rikli DA Regazzoni P. Fractures of the distal radius treated by internal fixation and early function. A prospective study of 73 consecutive patients. J Bone Joint Surg Br. 2000;82:340–4. 10.1302/0301-620x.82b3.10099 10813166

[6] Jupiter JB Fernandez DL. Comparative classification for fractures of the distal end of the radius. J Hand Surg Am. 1997;22:563–71. 10.1016/S0363-5023(97)80110-4 9260608

[7] Tajima T Saito H. A new classification of distal radius fractures and corresponding treatment methods. Handchir Mikrochir Plast Chir. 1991;23:227–35. 1757004

[8] Saab M Guerre E Chantelot C. Contribution of arthroscopy to the management of intra-articular distal radius fractures: knowledge update based on a systematic 10-year literature review. Orthop Traumatol Surg Res. 2019;105:1617–25 10.1016/j.otsr.2019.06.016 31526708

[9] Iwamae M Yano K Kaneshiro Y Sakanaka H. Arthroscopic reduction of an irreducible distal radioulnar joint in Galeazzi fracture-dislocation due to a fragment of the ulnar styloid: a case report. BMC Musculoskelet Disord. 2019;20:354. 10.1186/s12891-019-2735-5 PMC6668188 31366350

[10] Aguirre GL Cristiani WM Robles C. Fracturas intraarticulares de radio distal: reducción bajo fluoroscopía versus asistencia artroscópica. Revisión sistemática [Distal radius intraarticular fractures: fluoroscopy reduction versus arthroscopic assistance. Systematic review]. Acta Ortop Mex. 2020;34:226–432. 34020525

[11] Meinberg EG Agel J Roberts CS Karam MD Kellam JF. Fracture and Dislocation Classification Compendium-2018. J Orthop Trauma. 2018;32(Suppl 1):S1–S170. 10.1097/BOT.0000000000001063 29256945

[12] García-González N Berumen-Nafarrate E Ávila-Carrasco L Martínez-Fierro M Monárrez-Espino J. Reliability of radiographic parameters to determine the surgical success in distal radius fractures. Acta Ortop Mex. 2022;36:216–22. 36977640

[13] Perugia D Guzzini M Civitenga C. Is it really necessary to restore radial anatomic parameters after distal radius fractures? Injury. 2014;45(Suppl 6):S21–6. 10.1016/j.injury.2014.10.018 25457314

[14] Murakami K Abe Y Takahashi K. Surgical treatment of unstable distal radius fractures with volar locking plates. J Orthop Sci. 2007;12:134–40. 10.1007/s00776-006-1103-0 17393268

[15] Wright TW Horodyski M Smith DW. Functional outcome of unstable distal radius fractures: ORIF with a volar fixed-angle tine plate versus external fixation. J Hand Surg Am. 2005;30:289–99. 10.1016/j.jhsa.2004.11.014 15781351

[16] Goldfarb CA Yin Y Gilula LA Fisher AJ Boyer MI. Wrist fractures: what the clinician wants to know. Radiology. 2001;219:11–28. 10.1148/radiology.219.1.r01ap1311 11274530

[17] Andersen DJ Blair WF Steyers CM Adams BD el-Khouri GY Brandser EA. Classification of distal radius fractures: an analysis of interobserver reliability and intraobserver reproducibility. J Hand Surg Am. 1996;21:574–82. 10.1016/s0363-5023(96)80006-2 8842946

[18] Yu F Pang B Liu Y. Wrist arthroscopy-assisted reduction for distal radius fracture and its associated injuries: clinical observation of triangular fibrocartilage complex injuries. Ann Ital Chir. 2024;95:78–90. 38469615

[19] Knirk JL Jupiter JB. Intra-articular fractures of the distal end of the radius in young adults. J Bone Joint Surg Am. 1986;68:647–59. 3722221

[20] Doi K Hattori Y Otsuka K Abe Y Yamamoto H. Intra-articular fractures of the distal aspect of the radius: arthroscopically assisted reduction compared with open reduction and internal fixation. J Bone Joint Surg Am. 1999;81:1093–110. 10.2106/00004623-199908000-00005 10466642

[21] Jupiter JB Fernandez DL Toh CL Fellmann T Ring D. Operative treatment of volar intra-articular fractures of the distal end of the radius. J Bone Joint Surg Am. 1996;78:1817–28. 10.2106/00004623-199612000-00004 8986658

[22] Catalano LW 3rd Barron OA Glickel SZ. Assessment of articular displacement of distal radius fractures. Clin Orthop Relat Res. 2004;423:79–84. 10.1097/01.blo.0000132884.51311.28 15232430

[23] Abe Y. Plate presetting and arthroscopic reduction technique (PART) for treatment of distal radius fractures. Handchir Mikrochir Plast Chir. 2014;46:278–85. 10.1055/s-0034-1387705 25290270

[24] Lindau T Arner M Hagberg L. Intraarticular lesions in distal fractures of the radius in young adults. A descriptive arthroscopic study in 50 patients. J Hand Surg Br. 1997;22:638–43. 10.1016/s0266-7681(97)80364-6 9752922

